# Artificial intelligence in psychiatry emergencies: Roles and challenges

**DOI:** 10.1192/j.eurpsy.2026.10939

**Published:** 2026-07-31

**Authors:** E. C. Ezema, B. Aribisala, A. Meftah, T. Olupona, T. Htet, T. Sultana

**Affiliations:** Psychiatry, One Brooklyn Health-Interfaith , Brooklyn, United States

## Abstract

**Introduction:**

The introduction of artificial intelligence (AI) has positively impacted many aspects of healthcare delivery. The rising global incidence of mental illness underscores the need for AI in psychiatry, where it assists in real-time mental health assessments. The patients’ visitation for psychiatric emergencies poses significant health, financial, and administrative burdens to hospitals. AI has helped spot impulsivity, hyperactivity, and suicidal thoughts or behaviors, thereby accelerating care provision for psychiatric emergencies, reducing these burdens. However, there are concerns about data inconsistencies, quality, and transparency; confabulated output and biases pose challenges.

**Objectives:**

To evauate how AI has positively impacted psychiatric emergency care delivery.

To evaluate the challenges.

**Methods:**

A systematic review using search terms like “AI and psychiatric emergency,” “Roles of AI in psychiatric emergency,” and “Challenges of AI in psychiatric emergencies” was conducted on PubMed, Google, and Google Scholar for peer-reviewed articles published up until September 2025.

The search yielded 73 articles, but further screening led to the final selection of 18 articles.

**Results:**

AI has the potential to advance psychiatric patient care through increased efficiency, quality, better access, more accurate diagnosis, guided prognostication, and the discovery of novel algorithmic systems. As researchers continue to explore more appropriate modalities to predict, classify, or prognosticate mental illnesses like acute depression and psychosis, or predict the risk of suicide. AI can scan posts for language and image patterns that might indicate suicidal thoughts or behaviors. By these measures, there is an enhanced immediate detection of psychiatric emergencies necessitating urgent interventions, especially with rising incidence of suicides.

Despite AI’s impact in reducing the need for human resources and costs and improving time efficiency, it still poses some notable challenges. Besides the ethical concern, other challenging considerations include concerns with patient-provider interactions, validation, privacy, security, biases, urban/rural divides, language hurdles, budgetary restraints, technological difficulties, training, and professional issues.

**Conclusions:**

Although AI has proven helpful in psychiatry, its relevance is still being discussed. A study argued that psychiatry mainly depends on human skills like empathy, understanding, and the therapeutic relationship between patient and psychiatrist, so AI is unlikely to completely replace psychiatrists. Despite the challenges, AI developments hold the potential to create a new futuristic world where psychiatrists use AI as an ally in prompt, accurate, and contextualized treatment.

**Disclosure of Interest:**

None Declared

